# Crystal structure, Hirshfeld surface analysis and contact enrichment ratios of 1-(2,7-di­methyl­imidazo[1,2-*a*]pyridin-3-yl)-2-(1,3-di­thio­lan-2-yl­idene)ethanone monohydrate

**DOI:** 10.1107/S2056989019015755

**Published:** 2019-11-29

**Authors:** Yvon Bibila Mayaya Bisseyou, Mahama Ouattara, Pénétjiligué Adama Soro, R. C. A. Yao-Kakou, Abodou Jules Tenon

**Affiliations:** a Laboratoire de Cristallographie et Physique Moléculaire, UFR des Sciences des Structures de la Matière et de Technologie, Université Félix Houphouët-Boigny, 01 BP V34 Abidjan, Côte d’Ivoire; b Département de Chimie Thérapeutique et Chimie Organique Pharmaceutique, UFR Sciences Pharmaceutiques et Biologiques, Université Félix Houphouët-Boigny, 01 BP V34 Abidjan, Côte d’Ivoire

**Keywords:** crystal structure, hybrid mol­ecule, Hirshfeld surface analysis, enrichment contact, hydrogen bond

## Abstract

In the title hydrated hybrid compound C_14_H_14_N_2_OS_2_·H_2_O, the planar imidazo[1,2-*a*]pyridine ring system is linked to the 1,3-di­thiol­ane moiety by an enone bridge. The atoms of the C—C bond in the 1,3-di­thiol­ane ring are disordered over two positions with occupancies of 0.579 (14) and 0.421 (14) and both disordered rings adopt a half-chair conformation. The oxygen atom of the enone bridge is involved in a weak intra­molecular C—H⋯O hydrogen bond, which generates an *S*(6) graph-set motif. In the crystal, the hybrid mol­ecules are associated in *R*_2_^2^(14) dimeric units by weak C—H⋯O inter­actions. O—H⋯O hydrogen bonds link the water mol­ecules, forming infinite self-assembled chains along the *b-*axis direction to which the dimers are connected *via* O—H⋯N hydrogen bonding. Analysis of inter­molecular contacts using Hirshfeld surface analysis and contact enrichment ratio descriptors indicate that hydrogen bonds induced by water mol­ecules are the main driving force in the crystal packing formation.

## Abstract 2

The synthesis of a hybrid mol­ecule is reported. The crystal structure of the monohydrate was investigated using Hirshfeld surface analysis and enrichment contact ratios. Hydrogen bonds induced by guest water mol­ecules are the main driving force in crystal packing formation.

## Chemical context   

The imidazo[1,2-*a*]pyridine ring system was described for the first time in 1925 (Chichibabin, 1925 ▸). Compounds with the imidazo[1,2-*a*]pyridine scaffold exhibit a plethora of biological activities, including acting as receptor ligands, anti-infectious agents, enzyme inhibitors *etc*. as well as being potential nitro­gen heterobicycle therapeutic agents, as described by recent studies (Goel *et al.*, 2016 ▸; Deep *et al.*, 2017 ▸; Kuthyala *et al.*, 2018 ▸). On the other hand, compounds containing the 1,3-di­thio­lan-2-yl­idene moiety have been found to exhibit valuable pharmacological activities, including use as potent broad-spectrum fungicides (Tanaka *et al.*, 1976 ▸, Wang *et al.*, 1994 ▸), anti­tumor agents (Huang *et al.*, 2009 ▸), potent cephalosporinase inhibitors (Ohya *et al.*, 1982 ▸) and anti-HIV agents (Nguyen-Ba *et al.*, 1999 ▸; Besra *et al.*, 2005 ▸). In light of the above, we have incorporated into our research into the design of new potentially bioactive compounds the currently attractive mol­ecular hybridization strategy, which consists of the combination of at least two pharmacophoric moieties of different bioactive substances to produce a new hybrid compound that is medically more effective than its individual components (Viegas-Junior *et al.* 2007 ▸; Meunier, 2008 ▸). Yang *et al.* (2012 ▸) have shown that this approach is an effective way to develop novel and potent drugs for different targets.
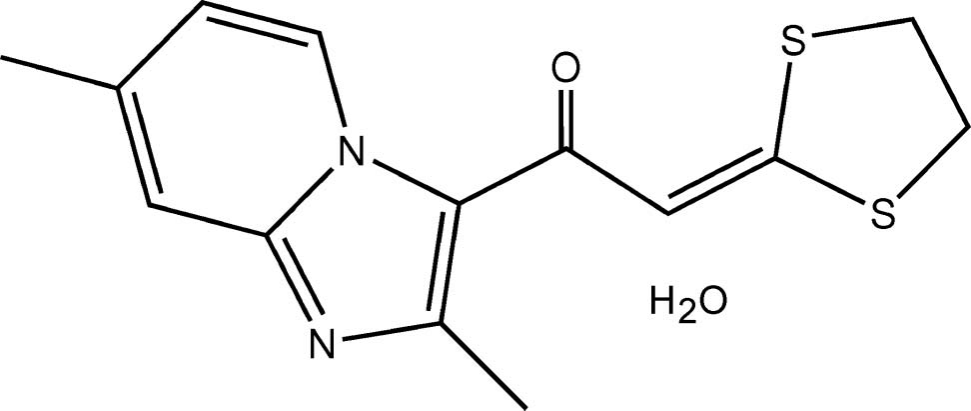


Herein we report the synthesis, crystal and mol­ecular structure of the title compound, an hybrid compound containing both imidazo[1,2-*a*]pyridine and 1,3-di­thiol­ane scaffolds. Moreover, since this compound crystallizes as a hydrate, the presence of water mol­ecules in the crystal structure is likely to alter its thermodynamic activity, which would impact its pharmacodynamic properties such as bioavailability and product performance (Khankari & Grant, 1995 ▸). From a crystallographic point of view, the intrusion of water mol­ecules into a solid state modifies the network of inter­molecular inter­actions between host mol­ecules by incorporating additional bonds between the organic host mol­ecules and water mol­ecules on the one hand, and between water mol­ecules on the other. To gain a better insight into the cohesive forces between host mol­ecules and intrusive water mol­ecules, and to highlight favored contacts likely to be the crystal driving force, an analysis of inter­molecular inter­actions was carried out using contact enrichment ratios (Jelsch *et al.*, 2014 ▸), a descriptor obtained from Hirshfeld surface analysis (Spackman & McKinnon, 2002 ▸), which allows an in-depth analysis of the atom–atom contacts in mol­ecular crystals, providing key information on their distribution and is a powerful tool for understanding the most important forces in inter­molecular inter­actions (Jelsch & Bibila Mayaya Bisseyou, 2017 ▸).

## Structural commentary   

**Figure 1:**
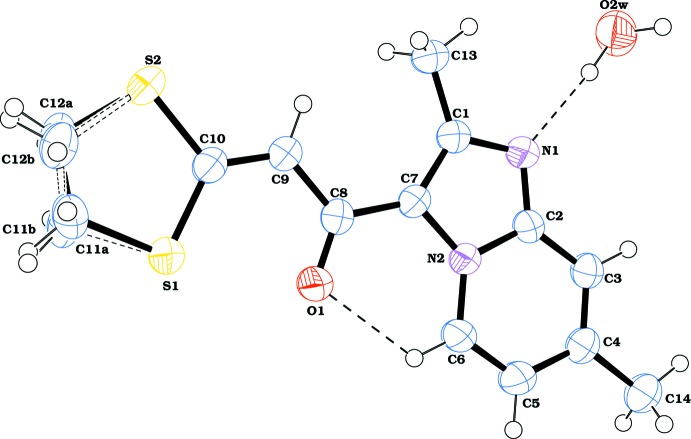
Mol­ecular structure of the title compound with atomic labelling. Displacement ellipsoids are drawn at the 50% probability level. H atoms are shown as spheres of arbitrary radius. The minor component of the disordered moiety is drawn with open bonds. Hydrogen bonds are shown as dashed lines.

**Figure 2:**
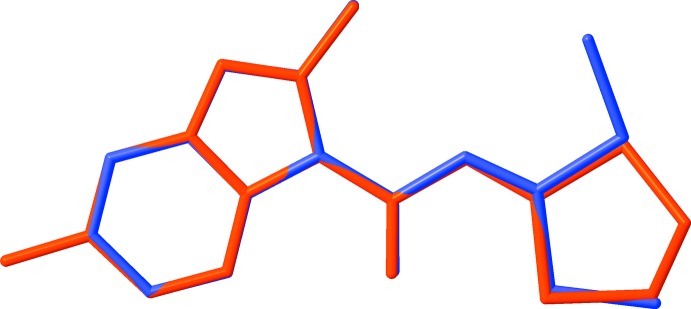
An overlay diagram of the title structure (red) with 1-(2-methyl­imidazo[1,2–*a*]pyridin-3-yl)-3,3-bis­(methyl­sulfan­yl)prop-2-enone structure (blue). H atoms and disordered moiety are excluded for clarity.

Fig. 1 ▸ shows the asymmetric unit of the title compound, which crystallizes as monohydrate in the ortho­rhom­bic space group *I*4_1_ *cd*. The hybrid mol­ecule consists of imidazo[1,2-*a*]pyridine and 1,3-di­thiol­ane scaffolds linked by an —CO—CH= enone bridge. The imidazo[1,2-*a*]pyridine ring system is essentially planar with a maximum deviation of 0.008 (1) Å for atom N1. Its geometrical parameters are similar to those found for 1-(2-methyl­imidazo[1,2-*a*]pyridin-3-yl)-3,3-bis­(methyl­sulfan­yl)prop-2-enone (Bibila Mayaya Bisseyou *et al.*, 2009 ▸), as illus­trated by the overlay of the structures shown in Fig. 2 ▸. In the 1,3-di­thiol­ane moiety, the C11 and C12 atoms of the C—C bond of the ring exhibit occupational disorder over two positions, with relative occupancies of 0.579 (14) and 0.421 (14) for the major and minor components, respectively. This disorder in the 1,3-di­thiol­ane skeleton is not uncommon and has been observed previously (Yang *et al.*, 2007 ▸; Liu *et al.*, 2008 ▸). Conformational analysis of the five-membered rings based on puckering parameters reveals a half-chair form for both disorder components [*Q*(2) = 0.419 (7)/0.443 (9) Å, φ(2) = 303.2 (9)/128.9 (11)° for the major and minor components, respectively]. The oxygen atom of the linker moiety is involved in a weak intra­molecular C6—H6⋯O1 hydrogen bond (Table 1 ▸), which generates an *S*(6) graph-set motif.

## Supra­molecular features   

**Figure 3:**
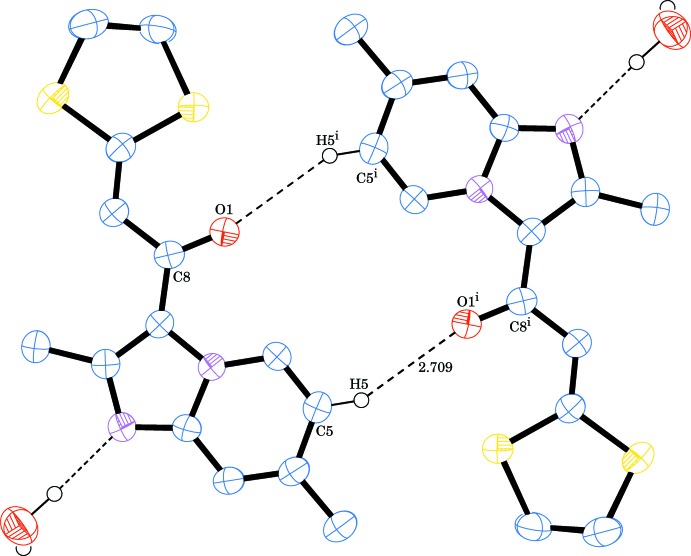
A partial packing diagram for the title compound showing the 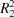
(14) graph-set motif generated by weak C—H⋯O hydrogen bonds plotted as dashed lines. H atoms not involved in the hydrogen bonding have been omitted for clarity.

**Figure 4:**
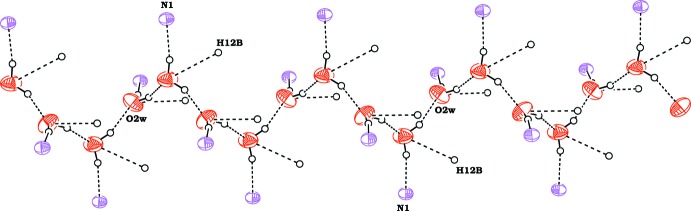
A view along *b* axis showing hydrogen-bonded self-assembled chain of water mol­ecules with the hydrogen bonds between the water and host mol­ecules shown as dashed lines. For clarity, the atoms in the host mol­ecules not involved in hydrogen bonds have been omitted.

**Figure 5:**
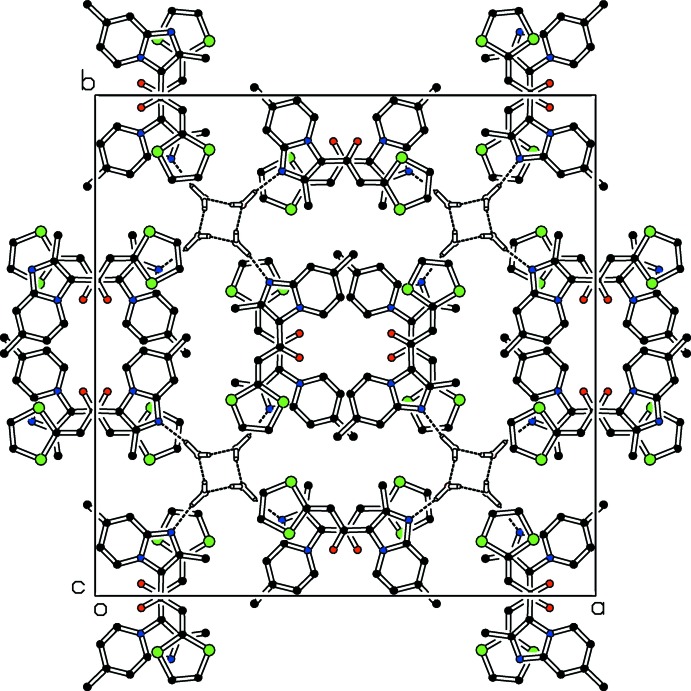
A view along the *c* axis of the crystal packing, showing the stacking of the host mol­ecules, with hydrogen bonds between water mol­ecules, and between water mol­ecules and host mol­ecules (dashed lines). For clarity, weak hydrogen contacts and some H atoms not involved in hydrogen bonding have been omitted.

In the crystal, the host mol­ecules form inversion dimers *via* pairwise weak C—H⋯O inter­actions [H5⋯O1^i^ = 2.71 Å; symmetry code as in Table 1 ▸, Fig. 3 ▸] with an 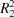
(14) ring motif. Salient inter­molecular inter­actions in the crystal packing are induced by the water mol­ecule. Each water mol­ecule is linked to two neighbouring water mol­ecules by O2*W*—H1*W*⋯O2*W* ^ii^ hydrogen bonds, generating an infinite self-assembled chain of water mol­ecules in a helical fashion along the *b* axis around which the host mol­ecules are linked *via* O2*W*—H2*W*⋯N1 hydrogen bonds and weak C12—H12*D*⋯O2*W* ^ii^ inter­actions (Fig. 4 ▸). The host mol­ecules are stacked on top of each other in alternating orientations along the *c-*axis direction (Fig. 5 ▸) and each is further involved in a cooperative contact with its adjacent homologue through a C—H⋯S inter­action (H5⋯S1^i^ = 3.00 Å).

## Hirshfeld surface analysis   

**Figure 6:**
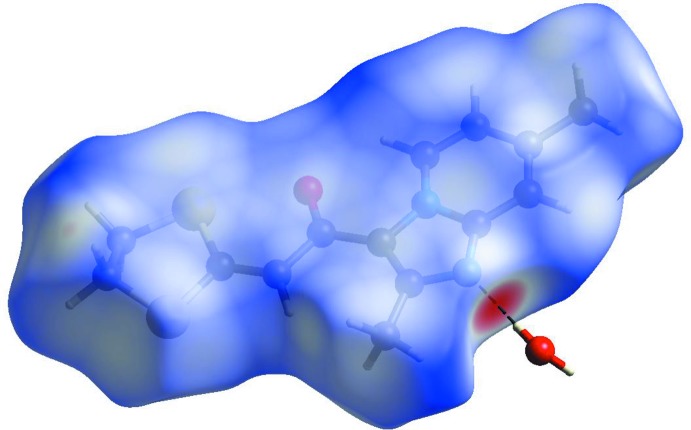
The three-dimensional Hirshfeld surface representation of the title compound plotted over *d*_norm_ in the range −0.5086 to 1.2492 a.u.

**Figure 8:**
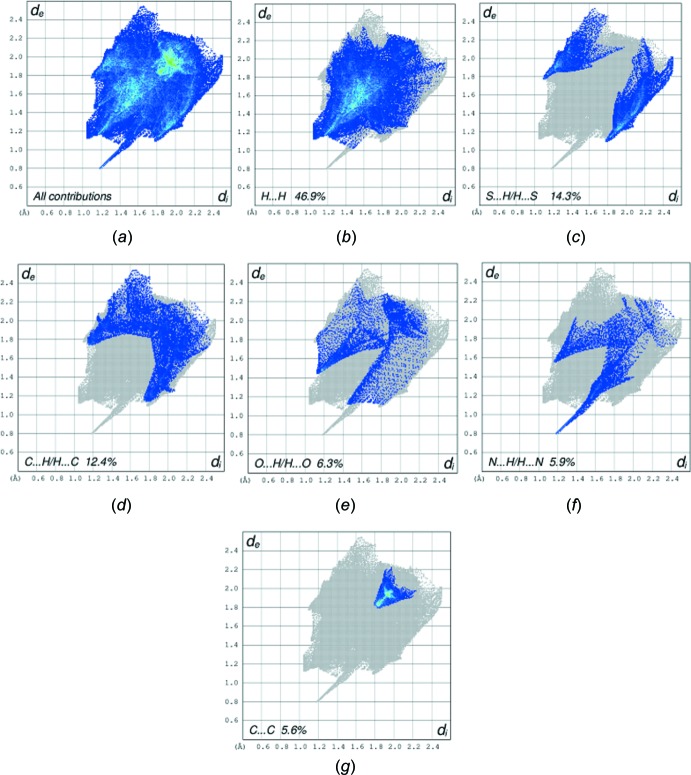
(*a*) The overall two-dimensional fingerprint plot for title compound and (*b*)–(*g*) those delineated into H⋯H, S⋯H/H⋯S, C⋯H/H⋯C, O⋯H/H⋯O, N⋯H/H⋯N, C⋯C/C⋯C contacts, respectively.

The Hirshfeld surface analysis (Spackman & Jayatilaka, 2009 ▸) and two-dimensional fingerprint plots (McKinnon *et al.*, 2007 ▸) were generated using *CrystalExplorer* (Turner *et al.*, 2017 ▸). The Hirshfeld surface (HS) mapped over *d*_norm_ in the range −0.5072 to 1.2974 a.u. and shape-index (range −1.0 to 1.0 a.u.) are displayed in Figs. 6 ▸ and 7 ▸, respectively. The red spot on the HS indicates the O2*W*—H2*W*⋯N1 hydrogen bond while the pale-red spot near H12*B* illustrates the weak C—H⋯O2*W* inter­action. The white spots represent H⋯O, H⋯S and H⋯H contacts. On the shape-index surface, convex blue regions indicate hydrogen-donor groups, while concave red regions indicate hydrogen-acceptor groups and S⋯N and S⋯C contacts and O⋯C inter­actions. The fingerprint plots show the contribution of different types of inter­molecular inter­actions (Fig. 8 ▸). The largest contribution (46.9%) is from the weak van der Waals H⋯H contacts, followed by S⋯H/H⋯S (14.3%), C⋯H/H⋯C (12.4%) and O⋯H/H⋯O (6.3%) inter­actions. The fingerprint plot for the N⋯H/H⋯N contacts (5.9% contribution) shows a sharp spike pointing toward the origin of the plot, which highlights the strong hydrogen-bonding between the host mol­ecule and water mol­ecule. The C⋯C contacts, with a V-shaped distribution of points, contribute 5.7%.

In order to detect favoured contacts and highlight the crystal driving force, enrichment ratios were computed with *MoProViewer* (Guillot *et al.*, 2014 ▸). The enrichment ratio *E_XY_* of a chemical element pair (*X*, *Y*) is defined as the ratio between the proportion of actual crystal contacts between the different chemical species (*X*, *Y*) and the theoretical proportion of random equiprobable contacts (Jelsch *et al.*, 2014 ▸). The asymmetric unit of the title compound is composed of two entities and in order to analyse all contacts present in the crystal, the host mol­ecule and a neighboring water mol­ecule not in contact each other were selected in order to obtain the integral Hirshfeld surfaces of each entity for the computation of the enrichment ratios. In addition, the hydro­phobic Hc atoms bound to carbon were distinguished from the more polar Ho water hydrogen atoms and oxygen atoms were also differentiated (O = ketone oxygen atom and O*W =* water oxygen atom). The results obtained are summarized in Table 2 ▸. The hydro­phobic Hc atoms, which constitute the largest part of the Hirshfeld surface, exhibit Hc⋯Hc self-contacts with an enrichment ratio equal to 1.0. The hydro­phobic C⋯Hc inter­actions are unprivileged with *E*_CHc_ = 0.76 and correspond to weak C—H⋯C inter­actions. These inter­actions are under-represented because competition with the S⋯Hc, O*W*⋯Hc and weak O⋯Hc hydrogen bonds, the first two of which appear favoured with enrichment values of 1.35 and 1.14, respectively, and the last slightly under-represented with an enrichment ratio of 0.98. The C⋯C contacts are privileged and display an enrichment value of 1.85, which highlight mol­ecules stacking one on top of the other as shown in Fig. 5 ▸. This type of stacking inter­action is generally favoured in heterocyclic compounds because of the favourable electrostatic complementary orientations of mol­ecules in the crystal packing. This result is in agreement to the findings reported by Jelsch *et al.* (2014 ▸). These stacking inter­actions induce N⋯S, O⋯C and S⋯C contacts displaying enrichment ratios of 1.58, 2.08 and 1.33, respectively. The N⋯Ho and O*W*⋯Ho polar contacts with the highest enrichment ratios of 5.03 and 5.19, respectively, are the most favoured contacts. These contacts correspond to the strong O2*W*—H2*W*⋯N1 and O2*W*—H1*W*⋯O2*W* hydrogen bonds (Table 1 ▸) observed in the crystal structure. Although crystallization is the result of concerted actions of all of the different inter­actions present within the crystal, the high enrichment value of the N⋯Ho and O*W*⋯Ho polar contacts reveal that these inter­molecular inter­actions are the main driving force in the crystal packing formation of the title compound.

## Database survey   

A search of the Cambridge Structural Database (WebCSD; Thomas *et al.*, 2010 ▸) gave 66 hits for structures having an imidazo[1,2-*a*]pyridin-3-yl moiety and 157 entries for structures containing an 1,3-di­thio­lan-2-yl­idene scaffold. No structure containing both fragments simultaneously has been determined to date. However, there is one imidazo[1,2-*a*]pyridin-3-yl derivative monohydrate that closely resembles the title compound *viz.* 1-(2-methyl­imidazo[1,2-*a*]pyridin-3-yl)-3,3-bis­(methyl­sulfan­yl)prop-2-∊none monohydrate (CSD refcode FOVROY; Bibila Mayaya Bisseyou *et al.*, 2009 ▸).

## Synthesis and crystallization   

1-(2,7-Di­methyl­imidazol[1,2-*a*]pyridin-3-yl)ethanone (6.2 mmol) was dissolved in distilled dimethyl sulfoxide (15 ml), and the carbon di­sulfide (1.1 molar equivalents, 6.82 mmol) was added. After cooling the mixture to 273 K, sodium hydride (2.5 molar equivalents, 15.5 mmol) was added. After stirring for 30 min. at 273 K, the mixture was stirred at ambient temperature for 4 h. The solution was then cooled at 273 K and 1,2-di­chloro ethane (2.5 molar equivalents, 15.5 mmol) was added dropwise. The resulting mixture was then stirred for 24 h and then poured into 50 ml of ice-cold water. The precipitate was filtered and recrystallized from a mixture of water–dioxane (2:1) to obtain brown single crystals of the title compound suitable for X-ray diffraction analysis (yield 76%; m.p. 453 K).

## Refinement   

Crystal data, data collection and structure refinement details are summarized in Table 3 ▸. Water H atoms were located in difference-Fourier maps and O*W*—H bond lengths were restrained to the target value of the neutron diffraction distance. All other H atoms were positioned geometrically (C—H = 0.93–0.97 Å) and were refined using a riding model with *U*_iso_(H) = 1.2*U*_eq_(C) or 1.5*U*_eq_(C-meth­yl). In the 1,3-di­thiol­ane ring, the carbon atoms of the C—C bond are disordered over two positions with refined occupancy factors of 0.579 (14) and 0.421 (14). C—C bond lengths in both disordered components were restrained to the target value of 1.513 Å (Allen *et al.*, 1987 ▸).

## Supplementary Material

Crystal structure: contains datablock(s) I. DOI: 10.1107/S2056989019015755/vm2224sup1.cif

Supporting information file. DOI: 10.1107/S2056989019015755/vm2224Isup2.cml

CCDC reference: 1967239

Additional supporting information:  crystallographic information; 3D view; checkCIF report

## Figures and Tables

**Figure 7:**
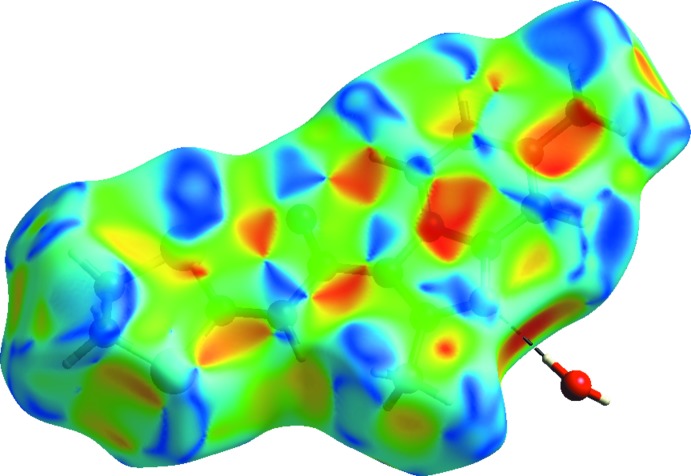
The three-dimensional Hirshfeld surface mapped over shape-index.

**Table 1:** Hydrogen-bond geometry (Å, °)

*D*—H⋯*A*	*D*—H	H⋯*A*	*D*⋯*A*	*D*—H⋯*A*
C6—H6⋯O1	0.93	2.24	2.812 (3)	119
O2*W*—H2*W*⋯N1	0.97 (1)	1.99 (2)	2.949 (3)	170 (6)
C5—H5⋯O1^i^	0.93	2.71	3.560 (3)	153
O2*W*—H1*W*⋯O2*W* ^ii^	0.97 (1)	1.92 (2)	2.837 (2)	157 (4)
C12*A*—H12*B*⋯O2*W* ^iii^	0.97	2.66	3.577 (11)	157

Symmetry codes: (i) 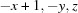; (ii) 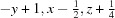; (iii) 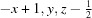.

**Table 2:** Inter­molecular contacts and enrichment ratios (%) on the Hirshfeld surface by atom type The top part of the table gives the surface contribution S*_*X*_* of each chemical type *X* to the Hirshfeld surface. The next part shows the percentage contributions C_*XY*_ of the actual contact types to the surface and the lower part of the table shows the E_*XY*_ enrichment contact ratios. *E*_*XY*_ ratios larger than unity are enriched contacts and those lower than unity are impoverished.

Atom type	Ho	C	N	O	S	Hc	Ow
Surface	7.70	23.26	3.69	2.77	14.46	43.96	4.17
							
Contact							
Ho							
C		9.40					
N	3.20						
O	0.00	3.00					
S	0.70	9.00	2.00				
Hc	8.40	15.00	2.40	3.00	17.60	18.70	
O*W*	3.50	0.00	0.00	0.00	0.00	4.10	0.00
							
Enrichment							
O*W*	5.19						
N	5.03						
S	0.27	1.33	1.58				
C	0.00	1.85	0.00	2.08			
Hc	1.19	0.76	0.72	0.98	1.35	1.00	1.14

**Table 3:** Experimental details

Crystal data	
Chemical formula	C_14_H_14_N_2_OS_2_·H2O
*M*_r_	308.41
Crystal system, space group	Tetragonal, *I*4_1_ *c* *d*
Temperature (K)	293
*a*, *c* (Å)	28.3247 (7), 7.2820 (2)
*V* (Å^3^)	5842.3 (3)
*Z*	16
Radiation type	Mo *K*α
μ (mm^−1^)	0.37
Crystal size (mm)	0.35 × 0.20 × 0.15

Data collection	
Diffractometer	Nonius KappaCCD
Absorption correction	Multi-scan (Blessing, 1995 ▸)
*T*_min_, *T*_max_	0.927, 0.963
No. of measured, independent and observed [*I* > 2σ(*I*)] reflections	22803, 3672, 2765
*R*_int_	0.044

Refinement	
*R*[*F* ^2^ > 2σ(*F* ^2^)], *wR*(*F* ^2^), *S*	0.036, 0.102, 1.04
No. of reflections	3672
No. of parameters	211
No. of restraints	43
H-atom treatment	H atoms treated by a mixture of independent and constrained refinement
Δρ_max_, Δρ_min_ (e Å^−3^)	0.22, −0.29
Absolute structure	Flack *x* determined using 1012 quotients [(*I* ^+^)−(*I* ^−^)]/[(*I* ^+^)+(*I* ^−^)] (Parsons *et al.* 2013 ▸)
Absolute structure parameter	−0.01 (4)

Computer programs: *COLLECT* (Nonius, 1997 ▸), *DENZO*/*SCALEPACK* (Otwinowski & Minor, 1997 ▸), *SHELXS97* (Sheldrick, 2008 ▸), *SHELXL2014* (Sheldrick, 2015 ▸), *ORTEP-3 for Windows* and *WinGX* (Farrugia, 2012 ▸), *PLATON* (Spek, 2009 ▸), *OLEX2* (Dolomanov *et al.*, 2009 ▸) and *publCIF* (Westrip, 2010 ▸).

## supplementary crystallographic information

### Crystal data

**Table:**

C_14_H_14_N_2_OS_2_·H2O	*D*_x_ = 1.403 Mg m^−^^3^
*M**_r_* = 308.41	Mo *K*α radiation, λ = 0.71073 Å
Tetragonal, *I*4_1_*c**d*	Cell parameters from 22217 reflections
*a* = 28.3247 (7) Å	θ = 1.4–30.0°
*c* = 7.2820 (2) Å	µ = 0.37 mm^−^^1^
*V* = 5842.3 (3) Å^3^	*T* = 293 K
*Z* = 16	Parallelepiped, brown
*F*(000) = 2592	0.35 × 0.20 × 0.15 mm

### Data collection

**Table:**

Nonius KappaCCD diffractometer	2765 reflections with *I* > 2σ(*I*)
phi and ω scan	*R*_int_ = 0.044
Absorption correction: multi-scan (Blessing, 1995)	θ_max_ = 30.0°, θ_min_ = 2.0°
*T*_min_ = 0.927, *T*_max_ = 0.963	*h* = −37→30
22803 measured reflections	*k* = −39→37
3672 independent reflections	*l* = −9→7

### Refinement

**Table:**

Refinement on *F*^2^	H atoms treated by a mixture of independent and constrained refinement
Least-squares matrix: full	*w* = 1/[σ^2^(*F*_o_^2^) + (0.0537*P*)^2^ + 1.6604*P*] where *P* = (*F*_o_^2^ + 2*F*_c_^2^)/3
*R*[*F*^2^ > 2σ(*F*^2^)] = 0.036	(Δ/σ)_max_ = 0.001
*wR*(*F*^2^) = 0.102	Δρ_max_ = 0.22 e Å^−^^3^
*S* = 1.04	Δρ_min_ = −0.29 e Å^−^^3^
3672 reflections	Extinction correction: SHELXL2014 (Sheldrick, 2015), Fc^*^=kFc[1+0.001xFc^2^λ^3^/sin(2θ)]^-1/4^
211 parameters	Extinction coefficient: 0.0024 (6)
43 restraints	Absolute structure: Flack *x* determined using 1012 quotients [(*I*^+^)-(*I*^-^)]/[(*I*^+^)+(*I*^-^)] (Parsons *et al.* 2013)
Hydrogen site location: mixed	Absolute structure parameter: −0.01 (4)

### Special details

**Table:**

Geometry. All esds (except the esd in the dihedral angle between two l.s. planes) are estimated using the full covariance matrix. The cell esds are taken into account individually in the estimation of esds in distances, angles and torsion angles; correlations between esds in cell parameters are only used when they are defined by crystal symmetry. An approximate (isotropic) treatment of cell esds is used for estimating esds involving l.s. planes.

### Fractional atomic coordinates and isotropic or equivalent isotropic displacement parameters (Å^2^)

**Table:**

	*x*	*y*	*z*	*U*_iso_*/*U*_eq_	Occ. (<1)
S1	0.38738 (2)	0.12434 (2)	0.85051 (16)	0.0539 (2)	
S2	0.39148 (2)	0.22768 (2)	0.85799 (17)	0.0626 (2)	
O1	0.47583 (6)	0.09202 (6)	0.8604 (5)	0.0651 (6)	
N2	0.57546 (6)	0.09070 (6)	0.8622 (5)	0.0402 (4)	
N1	0.62625 (6)	0.15130 (7)	0.8534 (4)	0.0419 (4)	
C1	0.58159 (8)	0.16831 (8)	0.8543 (6)	0.0413 (5)	
C2	0.62206 (7)	0.10414 (8)	0.8578 (5)	0.0392 (5)	
C3	0.65720 (8)	0.06930 (8)	0.8584 (5)	0.0445 (5)	
H3	0.6888	0.0781	0.8543	0.053*	
C4	0.64537 (8)	0.02258 (8)	0.8648 (6)	0.0474 (6)	
C5	0.59688 (9)	0.01044 (9)	0.8715 (6)	0.0559 (8)	
H5	0.5883	−0.0212	0.8775	0.067*	
C6	0.56270 (9)	0.04398 (9)	0.8694 (7)	0.0539 (7)	
H6	0.5310	0.0354	0.8727	0.065*	
C7	0.54822 (8)	0.13206 (7)	0.8609 (5)	0.0414 (5)	
C8	0.49691 (9)	0.13052 (7)	0.8601 (7)	0.0451 (5)	
C9	0.47020 (8)	0.17414 (8)	0.8611 (6)	0.0480 (6)	
H9	0.4863	0.2028	0.8650	0.058*	
C10	0.42253 (8)	0.17425 (8)	0.8563 (6)	0.0432 (5)	
C11A	0.3325 (3)	0.1573 (3)	0.8856 (14)	0.0577 (19)	0.579 (14)
H11A	0.3064	0.1402	0.8309	0.069*	0.579 (14)
H11B	0.3264	0.1604	1.0161	0.069*	0.579 (14)
C12A	0.3362 (3)	0.2057 (3)	0.7998 (15)	0.0585 (18)	0.579 (14)
H12A	0.3115	0.2262	0.8466	0.070*	0.579 (14)
H12B	0.3330	0.2035	0.6674	0.070*	0.579 (14)
C11B	0.3315 (4)	0.1526 (4)	0.792 (2)	0.054 (2)	0.421 (14)
H11C	0.3288	0.1570	0.6607	0.065*	0.421 (14)
H11D	0.3051	0.1337	0.8344	0.065*	0.421 (14)
C12B	0.3324 (4)	0.1995 (4)	0.8896 (19)	0.058 (3)	0.421 (14)
H12C	0.3262	0.1950	1.0194	0.069*	0.421 (14)
H12D	0.3080	0.2199	0.8400	0.069*	0.421 (14)
C13	0.57437 (9)	0.22043 (8)	0.8531 (7)	0.0545 (6)	
H13A	0.6044	0.2360	0.8444	0.082*	
H13B	0.5589	0.2299	0.9644	0.082*	
H13C	0.5552	0.2290	0.7496	0.082*	
C14	0.68205 (9)	−0.01559 (9)	0.8647 (6)	0.0587 (7)	
H14A	0.7128	−0.0017	0.8536	0.088*	
H14B	0.6766	−0.0364	0.7629	0.088*	
H14C	0.6802	−0.0331	0.9773	0.088*	
O2W	0.70594 (8)	0.21823 (9)	0.8393 (5)	0.0750 (7)	
H1W	0.7256 (13)	0.2168 (16)	0.948 (4)	0.097 (15)*	
H2W	0.6805 (13)	0.1956 (14)	0.859 (9)	0.131 (19)*	

### Atomic displacement parameters (Å^2^)

**Table:**

	*U*^11^	*U*^22^	*U*^33^	*U*^12^	*U*^13^	*U*^23^
S1	0.0382 (3)	0.0429 (3)	0.0806 (5)	0.0006 (2)	0.0015 (4)	0.0037 (5)
S2	0.0479 (4)	0.0417 (3)	0.0982 (6)	0.0122 (3)	−0.0040 (5)	0.0009 (5)
O1	0.0367 (9)	0.0391 (9)	0.1197 (18)	−0.0002 (7)	−0.0006 (14)	−0.0017 (14)
N2	0.0334 (9)	0.0334 (9)	0.0539 (11)	0.0013 (7)	−0.0004 (12)	−0.0002 (12)
N1	0.0379 (10)	0.0359 (10)	0.0518 (11)	−0.0018 (7)	−0.0011 (13)	0.0002 (13)
C1	0.0394 (12)	0.0370 (11)	0.0474 (13)	−0.0004 (9)	−0.0002 (16)	0.0010 (15)
C2	0.0327 (11)	0.0389 (11)	0.0460 (13)	−0.0011 (8)	−0.0006 (14)	−0.0010 (15)
C3	0.0341 (11)	0.0439 (12)	0.0555 (14)	0.0026 (9)	0.0009 (14)	−0.0031 (15)
C4	0.0396 (12)	0.0434 (12)	0.0591 (16)	0.0080 (9)	−0.0002 (16)	−0.0025 (15)
C5	0.0454 (13)	0.0365 (13)	0.086 (2)	0.0029 (9)	0.0000 (19)	−0.002 (2)
C6	0.0356 (12)	0.0372 (12)	0.089 (2)	−0.0026 (9)	0.0004 (17)	0.0012 (17)
C7	0.0361 (11)	0.0348 (10)	0.0532 (14)	0.0037 (8)	−0.0006 (14)	0.0014 (15)
C8	0.0355 (10)	0.0414 (10)	0.0583 (14)	0.0024 (10)	−0.0007 (13)	0.000 (2)
C9	0.0378 (11)	0.0371 (11)	0.0692 (16)	0.0029 (9)	−0.0037 (17)	0.0002 (15)
C10	0.0401 (11)	0.0391 (11)	0.0504 (13)	0.0050 (10)	0.0012 (18)	0.0013 (15)
C11A	0.035 (2)	0.062 (3)	0.075 (5)	−0.001 (2)	0.003 (4)	−0.006 (4)
C12A	0.042 (3)	0.067 (4)	0.066 (4)	0.012 (3)	−0.005 (4)	−0.003 (3)
C11B	0.035 (4)	0.062 (4)	0.066 (5)	0.010 (3)	0.006 (5)	−0.003 (5)
C12B	0.043 (4)	0.060 (5)	0.070 (6)	0.012 (3)	0.009 (4)	−0.006 (5)
C13	0.0470 (13)	0.0363 (12)	0.0802 (19)	0.0001 (10)	−0.0017 (18)	0.0023 (18)
C14	0.0458 (13)	0.0488 (14)	0.082 (2)	0.0125 (10)	0.000 (2)	−0.005 (2)
O2W	0.0606 (13)	0.0720 (14)	0.092 (2)	−0.0131 (11)	−0.0092 (16)	0.0270 (17)

### Geometric parameters (Å, º)

**Table:**

S1—C10	1.729 (3)	C1—C7	1.396 (3)
S1—C11B	1.824 (11)	C1—C13	1.491 (3)
S1—C11A	1.831 (8)	C2—C3	1.402 (3)
S2—C12A	1.739 (8)	C3—C4	1.366 (3)
S2—C10	1.750 (2)	C4—C5	1.417 (3)
S2—C12B	1.868 (12)	C4—C14	1.500 (3)
O1—C8	1.243 (3)	C5—C6	1.356 (3)
N2—C6	1.373 (3)	C7—C8	1.454 (3)
N2—C2	1.374 (3)	C8—C9	1.449 (3)
N2—C7	1.403 (3)	C9—C10	1.351 (3)
N1—C2	1.342 (3)	C11A—C12A	1.509 (9)
N1—C1	1.354 (3)	C11B—C12B	1.506 (10)
			
C10—S1—C11B	98.4 (3)	C5—C4—C14	119.8 (2)
C10—S1—C11A	93.9 (3)	C6—C5—C4	121.4 (2)
C12A—S2—C10	98.1 (3)	C5—C6—N2	119.2 (2)
C10—S2—C12B	94.7 (4)	C1—C7—N2	104.01 (19)
C6—N2—C2	121.39 (19)	C1—C7—C8	134.3 (2)
C6—N2—C7	131.3 (2)	N2—C7—C8	121.7 (2)
C2—N2—C7	107.28 (19)	O1—C8—C9	119.8 (2)
C2—N1—C1	105.76 (18)	O1—C8—C7	120.4 (2)
N1—C1—C7	111.77 (19)	C9—C8—C7	119.8 (2)
N1—C1—C13	118.7 (2)	C10—C9—C8	121.6 (2)
C7—C1—C13	129.5 (2)	C9—C10—S1	125.03 (19)
N1—C2—N2	111.18 (19)	C9—C10—S2	120.27 (19)
N1—C2—C3	129.7 (2)	S1—C10—S2	114.69 (14)
N2—C2—C3	119.2 (2)	C12A—C11A—S1	110.3 (6)
C4—C3—C2	120.5 (2)	C11A—C12A—S2	106.6 (6)
C3—C4—C5	118.3 (2)	C12B—C11B—S1	105.2 (9)
C3—C4—C14	121.9 (2)	C11B—C12B—S2	109.6 (7)

### Hydrogen-bond geometry (Å, º)

**Table:**

*D*—H···*A*	*D*—H	H···*A*	*D*···*A*	*D*—H···*A*
C6—H6···O1	0.93	2.24	2.812 (3)	119
O2*W*—H2*W*···N1	0.97 (1)	1.99 (2)	2.949 (3)	170 (6)
C5—H5···O1^i^	0.93	2.71	3.560 (3)	153
O2*W*—H1*W*···O2*W*^ii^	0.97 (1)	1.92 (2)	2.837 (2)	157 (4)
C12*A*—H12*B*···O2*W*^iii^	0.97	2.66	3.577 (11)	157

Symmetry codes: (i) −*x*+1, −*y*, *z*; (ii) −*y*+1, *x*−1/2, *z*+1/4; (iii) −*x*+1, *y*, *z*−1/2.
